# Accurate Clinical Entity Recognition and Code Mapping of Anatomopathological Reports Using BioClinicalBERT Enhanced by Retrieval-Augmented Generation: A Hybrid Deep Learning Approach

**DOI:** 10.3390/bioengineering13010030

**Published:** 2025-12-27

**Authors:** Hamida Abdaoui, Chamseddine Barki, Ismail Dergaa, Karima Tlili, Halil İbrahim Ceylan, Nicola Luigi Bragazzi, Andrea de Giorgio, Ridha Ben Salah, Hanene Boussi Rahmouni

**Affiliations:** 1 Laboratory of Biophysics and Medical Technologies, Higher Institute of Medical Technologies of Tunis (ISTMT), University of Tunis El Manar, Tunis 1006, Tunisia; hamidaabdaoui53@gmail.com (H.A.); chamseddine.barki@istmt.utm.tn (C.B.); ridha.bensalah@istmt.utm.tn (R.B.S.); hanene.boussi@istmt.utm.tn (H.B.R.); 2 Higher Institute of Sport and Physical Education of Ksar Said, University of Manouba, Manouba 2010, Tunisia; phd.dergaa@gmail.com; 3 Department of Pathology, Faculty of Medicine of Tunis, Military Hospital of Tunis, Tunis 1008, Tunisia; tlili.karima@yahoo.fr; 4 Physical Education of Sports Teaching Department, Faculty of Sports Sciences, Atatürk University, 25240 Erzurum, Türkiye; 5 Laboratory for Industrial and Applied Mathematics (LIAM), Department of Mathematics and Statistics, York University, Toronto, ON M3J 1P3, Canada; 6 Artificial Engineering, 80121 Naples, Italy; andrea@degiorgio.info; 7 The Computer Science Research Centre, The University of the West of England, Bristol BS16 1QY, UK

**Keywords:** anatomopathological report, BioClinicalBERT, code mapping, deep learning, ICD-11, LOINC, named entity recognition, natural language processing, SNOMED CT, transformer architecture

## Abstract

Background: Anatomopathological reports are largely unstructured, which limits automated data extraction, interoperability, and large-scale research. Manual extraction and standardization are costly and difficult to scale. Objective: We developed and evaluated an automated pipeline for entity extraction and multi-ontology normalization of anatomopathological reports. Methods: A corpus of 560 reports from the Military Hospital of Tunis, Tunisia, was manually annotated for three entity types: sample type, test performed, and finding. The entity extraction utilized BioBERT v1.1, while the normalization combined BioClinicalBERT multi-label classification with retrieval-augmented generation, incorporating both dense and BM25 sparse retrieval over SNOMED CT, LOINC, and ICD-11. The performance was measured using precision, recall, F1-score, and statistical tests. Results: BioBERT achieved high extraction performance (F1: 0.97 for the sample type, 0.98 for the test performed, and 0.93 for the finding; overall 0.963, 95% CI: 0.933–0.982), with low absolute errors. For terminology mapping, the combination of BioClinicalBERT and dense retrieval outperformed the standalone and BM25-based approaches (macro-F1: 0.6159 for SNOMED CT, 0.9294 for LOINC, and 0.7201 for ICD-11). Cohen’s Kappa ranged from 0.7829 to 0.9773, indicating substantial to near-perfect agreement. Conclusions: The pipeline provides robust automated extraction and multi-ontology coding of anatomopathological entities, supporting transformer-based named entity recognition with retrieval-augmented generation. However, given the limitations of this study, multi-institutional validation is needed before clinical deployment.

## 1. Introduction

Anatomic pathology provides definitive histopathological assessments essential for disease classification, prognostic stratification, and treatment planning across oncology, infectious diseases, and inflammatory conditions [1,2]. Cancer accounts for approximately 10 million deaths annually worldwide, with anatomopathological diagnosis serving as the reference standard for tumor classification according to the World Health Organization (WHO) criteria [3]. Contemporary pathology workflows generate substantial volumes of narrative reports documenting macroscopic descriptions, microscopic findings, and diagnoses, which are integrated into electronic medical records [4,5]. These textual data support clinical decision-making, quality assurance, epidemiological surveillance, and retrospective research initiatives across healthcare institutions [6,7]. However, the predominance of unstructured free-text formats creates substantial barriers to automated information extraction, semantic interoperability, and large-scale computational analysis [8,9].

Converting free-text clinical narratives into structured, usable data is a key focus in the field of artificial intelligence (AI), which is rapidly transforming healthcare with applications for diagnostic imaging, predictive analytics, and clinical decision support. In specialized domains, AI methodologies have demonstrated significant utility, from systematic reviews of diagnostic criteria in endocrinology, such as for polycystic ovary syndrome [10], to novel neural networks that enhance tumor identification in oncology imaging [11]. A pivotal yet underexplored application is the semantic processing and standardization of unstructured clinical text. This work addresses that gap by focusing on anatomic pathology reports, where AI can automate the labor-intensive bottleneck of coding, thereby enabling data interoperability for research and clinical workflows.

The transformation of unstructured clinical narratives into structured, standardized data representations requires sophisticated natural language processing approaches capable of medical domain adaptation [12,13]. Named entity recognition has emerged as a foundational technique for identifying clinical concepts within free text, with transformer-based architectures demonstrating substantial performance gains over traditional rule-based approaches and statistical methods [14,15]. BioBERT introduced domain-specific pre-training on PubMed abstracts and PubMed Central (PMC) full-text articles, achieving state-of-the-art results across multiple biomedical named entity recognition benchmarks, including disease mentions, chemical compounds, and gene identifiers [16]. Subsequent models, including ClinicalBERT and BioClinicalBERT, incorporated clinical notes from the MIMIC-III database, further improving the performance on clinical entity extraction tasks [17,18]. These transformer architectures use self-attention mechanisms to capture long-range contextual dependencies and semantic relationships, which are essential for disambiguating medical terminology [19].

Beyond entity extraction, normalization to standard medical terminologies represents an equally critical challenge for achieving semantic interoperability across healthcare systems [20,21]. The Systematized Nomenclature of Medicine Clinical Terms (SNOMED CT) provides comprehensive coverage with over 350,000 active concepts spanning clinical findings, procedures, body structures, and organisms [22]. The Logical Observation Identifiers Names and Codes (LOINC) standardizes laboratory test nomenclature with approximately 95,000 terms, facilitating data exchange across laboratory information systems [23]. The International Classification of Diseases 11th Revision (ICD-11) offers a contemporary coding framework supporting epidemiological reporting, clinical documentation, and healthcare resource allocation [24]. However, mapping free-text clinical entities to these standardized terminologies requires sophisticated approaches capable of handling synonyms, abbreviations, contextual variations, and hierarchical concept relationships [25,26]. Recent research has explored metric learning, retrieval-based methods, and graph neural networks for entity linking tasks, with varied results depending on the complexity of the terminology and the availability of training data [27,28]. Retrieval-augmented generation has emerged as a promising paradigm that grounds language model predictions in external knowledge bases through dense vector retrieval, demonstrating improvements in factual accuracy and domain-specific question answering [29,30].

Despite these advances, several research gaps persist in automated pathology report processing. First, most existing studies focus on English-language clinical texts, with limited validation on non-English pathology reports from diverse geographic regions [31]. Second, multi-ontology mapping approaches typically evaluate the performance on individual terminologies rather than simultaneous mapping to SNOMED CT, LOINC, and ICD-11 within unified frameworks [32]. Third, the integration of retrieval-augmented approaches with supervised classification for medical entity normalization remains underexplored compared to standalone architectures [33]. Finally, rigorous statistical validation, including effect size reporting, confidence interval estimation, and inter-model comparison testing, is often absent from published studies [34].

Based on the identified research gaps, our study aimed to (i) develop and validate a BioBERT-based named entity recognition model for extracting the sample type, test performed, and finding entities from anatomopathological reports; (ii) implement a hybrid standardization framework combining BioClinicalBERT multi-label classification with dense retrieval-augmented generation for simultaneous mapping to the SNOMED CT, LOINC, and ICD-11 terminologies; and (iii) conduct a comprehensive performance evaluation with rigorous statistical testing, including bootstrap confidence intervals, agreement measures, and inter-model comparisons on real-world clinical data.

## 2. Materials and Methods

### 2.1. Ethical Approval

This retrospective study received approval from the Ethics Committee of the Military Hospital of Tunis, Tunis, Tunisia (decision number 116/2025/CLPP/Hôpital Militaire de Tunis) on 7 July 2025, before data collection and analysis. The study was conducted in accordance with the Declaration of Helsinki. All patient information was rigorously anonymized to ensure confidentiality, privacy, and data protection. Data handling and analysis were undertaken solely by anatomical pathologists and data scientists involved in the study, ensuring strict adherence to ethical standards and institutional guidelines.

### 2.2. Data Collection and Corpus Development

We collected 560 anatomopathological reports retrospectively from the information system of the Main Military Teaching Hospital of Tunis between January 2020 and December 2023. Each report corresponded to a unique patient case and was manually annotated by board-certified anatomical pathologists. Standard preprocessing included removing uninformative headers and footers, de-identifying sensitive personal data, and correcting only typographical errors to achieve complete anonymization. The annotation process identified three primary entity types: sample type (tissue specimens submitted for examination), test performed (histological techniques and staining methods), and finding (diagnostic observations and pathological descriptions).

To ensure consistent and reproducible annotation, we developed the following guidelines, as detailed in Table 1.

Manual assignment of the reference codes from SNOMED CT, LOINC, and ICD-11 terminologies was performed using official terminology resources obtained from https://www.snomed.org (accessed 15 August 2025), https://loinc.org (accessed 15 August 2025), and https://icd.who.int (accessed 15 August 2025), respectively.

### 2.3. External Knowledge Base Construction

For the retrieval-augmented generation component, we constructed an external knowledge base using the official releases of SNOMED CT (version 08.2025), LOINC (version 2.81), and ICD-11 (version 01/2024). To ensure clinical relevance to anatomical pathology, we selected relevant subsets from each terminology, guided by their organizational structures: hierarchies for SNOMED CT, class designations for LOINC, and chapter classifications for ICD-11. We integrated and indexed these standardized clinical concepts, along with their associated codes, labels, and relationships, to enable semantic similarity retrieval during model inference.

### 2.4. Named Entity Recognition Model

We implemented BioBERT v1.1 (dmis-lab/biobert-v1.1) for token-level entity classification, using the Hugging Face Transformers library, version 4.45.0 (Hugging Face Inc., New York, NY, USA) [16]. Reports were preprocessed and converted to JSON format with sentence-level segmentation. The BioBERT tokenizer applied WordPiece subword tokenization, with the token labels aligned to the subwords using word_ids() mapping. Filler tokens and unaligned tokens were masked using −100 label identifiers. The architecture consisted of bidirectional transformer layers generating contextualized feature vectors for each token position. Self-attention mechanisms captured the contextual dependencies across sequences according to the formulation [15]:

(1) $$Attention\left(Q,K,V\right)=\mathrm{softmax}(\frac{{QK}^{T}}{\sqrt{dₖ}})V$$

where Q,K, and V represent query, key, and value matrices, respectively, and dₖ denotes key vector dimensionality. A fully connected classification layer mapped contextualized embeddings to entity type predictions through softmax activation:

(2) $$P(y_{i}|x)=\frac{e^{z_{i}}}{\sum_{j}e^{z_{j}}}$$

where $z_{i}$ represents the logit score for class i. Model training optimized the cross-entropy loss:

(3) $$L=-\sum_{i}y_{i}\log\hat{y_{i}}$$

where $y_{i}$ denotes accurate labels and $\hat{y_{i}\;}\;$ represents predicted probabilities.

### 2.5. Training Configuration for Named Entity Recognition

BioBERT v1.1 was fine-tuned for three epochs using the AdamW optimizer as implemented in PyTorch, version 2.4.1 (PyTorch Foundation, San Francisco, CA, USA), with a learning rate of 2 × 10^−5^, a weight decay of 0.01, and a batch size of 8 for both the training and evaluation phases [35]. Cross-entropy loss with ignored labels set to −100 guided optimization. Data were split into 80% for training and 20% for testing. Training was conducted using Python 3.12.12 (Python Software Foundation, Wilmington, DE, USA) with the Hugging Face Transformers library and PyTorch, via the Hugging Face Trainer API, on NVIDIA GPU hardware/infrastructure (NVIDIA Corporation, Santa Clara, CA, USA). The evaluation metrics included the token-level precision, recall, and F1-score calculated separately for each entity class.

### 2.6. Entity Standardization Architecture

The standardization pipeline integrated supervised classification and semantic retrieval components. BioClinicalBERT from emilyalsentzer/Bio_ClinicalBERT encoded the extracted entity mentions into dense representations, projecting them into the model’s latent space [18]. A multi-label classification head produced probability distributions over the target code spaces for the SNOMED CT, LOINC, and ICD-11 terminologies. The top-k candidates with the highest probabilities were extracted from the classification outputs.

### 2.7. Retrieval-Augmented Generation Module

For retrieval augmentation, we encoded all the terminology concepts from the SNOMED CT, LOINC, and ICD-11 databases using BioClinicalBERT to generate dense vector representations. During inference, the query entity embeddings were compared with the indexed terminology vectors using the cosine similarity. The nearest-neighbor concepts were retrieved as candidate codes from the external knowledge base via dense passage retrieval [29].

### 2.8. Fusion and Reranking Strategy

The final standardization decision combined outputs from the BioClinicalBERT classification and retrieval modules through a learned fusion mechanism. A weighted decision rule was optimized via 5-fold cross-validation, determining whether to select the classifier’s prediction or the retrieved concept. When the classification and retrieval outputs disagreed, the system applied the learned weights to prioritize the more reliable component. As a fallback mechanism, retrieval-based predictions overrode classifier errors when the classification confidence was below the threshold values. This approach balanced discriminative accuracy from supervised learning with semantic robustness from knowledge base retrieval.

### 2.9. Training Configuration for Standardization

BioClinicalBERT was trained using the AdamW optimizer with cross-entropy loss on the annotated dataset for 10 epochs, with early stopping based on the validation loss. The retrieval-augmented generation component operated only during inference, retrieving the closest terminology codes based on the cosine similarity scores. The fusion module combined predictions from trained BioClinicalBERT and reference retrieval-augmented generation using weighted scoring, with the optimal weights determined through cross-validation on the training set. Final predictions for the SNOMED CT, LOINC, and ICD-11 codes were generated through the integrated fusion architecture.

### 2.10. Statistical Analysis

The performance evaluation employed precision (positive predictive value), recall (sensitivity), and F1-score (harmonic mean of the precision and recall), calculated separately for each entity type and terminology. Bootstrap resampling with 10,000 iterations estimated the 95% confidence intervals (CIs) for the performance metrics [36]. One-sample *t*-tests assessed whether the model performance significantly exceeded a baseline threshold of 0.5. Cohen’s Kappa and the Matthews correlation coefficient quantified the agreement between the predictions and ground truth annotations, accounting for chance agreement [37]. McNemar’s test evaluated the prediction discordance between model pairs [38]. Permutation tests with 10,000 iterations assessed whether the observed differences in accuracy between models could be due to chance. All the statistical analyses were conducted using Python 3.12.12 and its scientific computing libraries (NumPy version 2.1.1, SciPy version 1.14.1, scikit-learn version 1.5.2) on the held-out test set.

## 3. Results

### 3.1. Named Entity Recognition Performance by Entity Type

The BioBERT model achieved strong performance across all the clinical entity types on the test set. Table 2 presents the performance metrics organized by entity type, with an overall mean precision of 0.969, a recall of 0.958, and an F1-score of 0.963. Sample type entities achieved a precision of 0.97, a recall of 0.98, and an F1-score of 0.97. Test performed entities demonstrated precision of 0.97, a recall of 0.99, and an F1-score of 0.98. Finding entities achieved a precision of 0.97, a recall of 0.90, and an F1-score of 0.93.

### 3.2. Confusion Matrix Analysis

**Figure 1:**
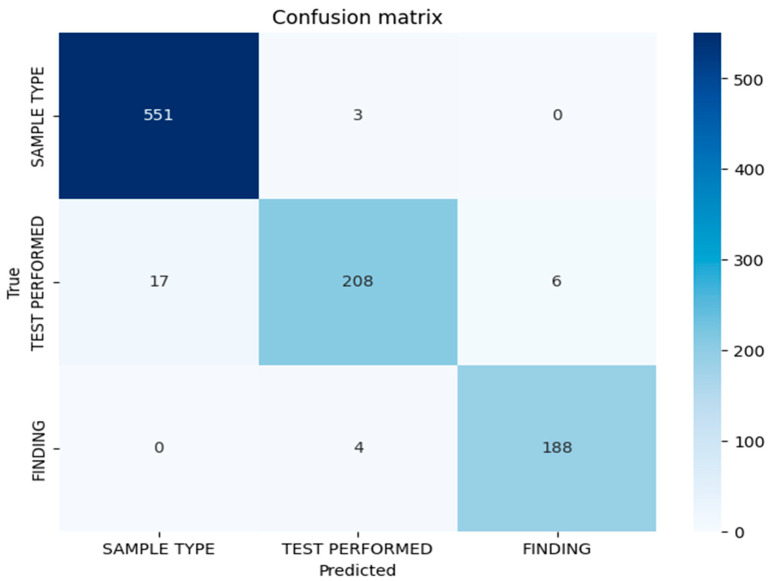
Confusion matrix of the BioBERT model on the test set. The values represent the number of tokens correctly or incorrectly classified for each entity.

Figure 1 represents the confusion matrix displaying the token-level classification results for the BioBERT model on the test set. The matrix demonstrates strong discrimination between entity classes, with minimal misclassification. The diagonal elements indicate correct classifications for each entity type, while the off-diagonal elements indicate misclassifications. The misclassification patterns reveal that the errors are primarily concentrated in the test performed entity category, suggesting lexical or contextual ambiguities in the procedural nomenclature descriptions in anatomopathological reports. The confusion matrix confirms the low cross-category confusion, indicating that the model effectively learns semantic distinctions between sample types, performed tests, and diagnostic findings.

### 3.3. Error Analysis by Entity Type

Table 3 presents the absolute and relative errors per entity for the BioBERT model. Test performed entities exhibited the highest error rate, with 16 misclassified tokens, resulting in a relative error rate of 6.9%. Sample type entities demonstrated a moderate level of difficulty, with 14 misclassified tokens and a relative error rate of 2.5%, reflecting the variability in the descriptions of anatomical specimens across reports. Finding entities showed very low error levels, with only two misclassified tokens and a relative error rate of 1%, indicating that the diagnoses are relatively consistent and easier to identify within the corpus. The diversity of terms and abbreviations used to describe specimens and procedures contributes to the classification challenges, particularly for test performed entities, where standardized terminology exists alongside institutional variations and procedural synonyms.

### 3.4. Confidence Intervals for Performance Metrics

Bootstrap estimation with 10,000 samples generated 95% CIs confirming robust performance stability, as presented in Table 4. The precision demonstrated narrow confidence intervals (95% CI: 0.967–0.970), indicating high stability for this metric across the bootstrap resampling iterations. The recall exhibited slightly wider confidence intervals (95% CI: 0.900–0.995), reflecting variability in detecting certain entity instances, particularly for finding entities. The F1-score confidence intervals (95% CI: 0.933–0.982) showed intermediate width, balancing the stability of the precision with the variability of the recall measurements. The narrow intervals overall confirm that the observed performance represents stable model behavior rather than stochastic variation or overfitting to the particular test set composition.

### 3.5. Statistical Significance Testing Against Baseline

One-sample *t*-tests comparing the model metrics against a baseline of 0.5 yielded extremely high t-statistics, as shown in Table 5, with all the *p*-values below 0.001. The t-statistics for precision, recall, and F1-score were 612.029, 15.71, and 30.24, respectively, confirming that the observed performance significantly exceeded the baseline thresholds with very high confidence. The exceptionally high t-statistic for the F1-score was driven by both high mean performance and low variance across the three entity types. The very low *p*-values confirm that all the model metrics significantly exceed the 0.5 baseline, indicating that the observed performance is not due to chance. The t-statistics for precision, while very high, reflects the small entity sample size (n = 3), which amplifies test statistics when the variance is low. The recall and F1-score t-values remained elevated, with the magnitudes being more intuitive, reflecting slightly greater variability in these metrics across entities.

### 3.6. Descriptive Statistics Across Entity Labels

Table 6 presents the metric statistics by label, revealing consistent high performance across all the entity types. A mean precision of 0.969 and a standard deviation of 0.001 demonstrate minimal variability in the positive predictive value across the entity classes. A mean recall of 0.958 with a standard deviation of 0.050 indicates slightly higher variability, suggesting that certain entities, such as finding, pose greater detection challenges due to the lexical diversity and contextual variation in diagnostic descriptions. The mean F1-score of 0.963 with a standard deviation of 0.025 confirms the balanced performance between precision and recall. The low standard deviations indicate that the model consistently achieves high performance across all the labels, with stable precision and F1-scores throughout the entity taxonomy.

### 3.7. Multi-Ontology Standardization Descriptive Performance

Table 7 presents the descriptive performance metrics across four model configurations evaluated on 53 test samples: BioClinicalBERT only (without external knowledge), dense retrieval-augmented generation (transformer-based embeddings indexed with Facebook AI Similarity Search or FAISS), BioClinicalBERT + BM25 (sparse retrieval-augmented generation without dense embeddings), and our proposed Fusion/Reranker system (BioClinicalBERT + dense Retrieval-Augmented Generation).

For the SNOMED CT code mapping, BioClinicalBERT alone achieved an accuracy of 0.7547, a precision of 0.5881, a recall of 0.6667, and a macro-F1 of 0.6124. Dense-only retrieval-augmented generation demonstrated intermediate performance with an accuracy of 0.6981, a precision of 0.5833, a recall of 0.5960, and a macro-F1 of 0.5856. The configuration using BM25 sparse retrieval (BioClinicalBERT + BM25) showed reduced performance, with an accuracy of 0.5849, a precision of 0.5000, a recall of 0.4907, and a macro-F1 of 0.4944, highlighting the limitations of traditional sparse retrieval methods for this task. In contrast, our Fusion/Reranker system achieved the highest performance, with an accuracy of 0.7925, a precision of 0.6136, a recall of 0.6263, and a macro-F1 of 0.6159, representing an improvement over BioClinicalBERT alone.

For the LOINC classification, the performance was substantially higher across all the models, with Fusion/Reranker achieving an accuracy of 0.9811, a precision of 0.9216, a recall of 0.9412, and a macro-F1 of 0.9294. BioClinicalBERT alone showed an accuracy of 0.9434, a precision of 0.8284, a recall of 0.8824, and a macro-F1 of 0.8445. Dense-only retrieval-augmented generation performed exceptionally well, matching the accuracy of Fusion/Reranker at 0.9811, suggesting strong lexical matching for the LOINC codes. Further, it exhibited a precision of 0.9216, a recall of 0.9412, and a macro-F1 of 0.9284. The BM25 configuration achieved an accuracy of 0.9245, a precision of 0.7963, a recall of 0.8333, and a macro-F1 of 0.8056, demonstrating that while BM25 performs reasonably for LOINC, our dense retrieval approach provides a performance improvement.

For the ICD-11 mapping, Fusion/Reranker achieved an accuracy of 0.8491, a precision of 0.7115, a recall of 0.7500, and a macro-F1 of 0.7201, compared to BioClinicalBERT’s accuracy of 0.7925, precision of 0.6623, recall of 0.7171, and macro-F1 of 0.6772. Dense-only retrieval-augmented generation showed the same accuracy as BioClinicalBERT at 0.7925 but had a lower macro-F1 of 0.6583. The precision and recall yielded values of 0.6500 and 0.6875, respectively. The BM25 configuration achieved an accuracy of 0.8113 and a macro-F1 of 0.6880, showing modest improvement over BioClinicalBERT alone but remaining inferior to our Fusion/Reranker system, with a precision of 0.6813 and a recall of 0.7125.

These results demonstrate three key findings: (1) traditional sparse retrieval (BM25) degrades performance compared to BioClinicalBERT alone for SNOMED CT, highlighting its inadequacy for this task; (2) our dense retrieval approach consistently outperforms BM25 across all the terminologies; and (3) the Fusion/Reranker architecture provides the optimal combination, achieving consistent superiority across all three medical terminologies while emphasizing the critical importance of dense embeddings over sparse alternatives.

### 3.8. Agreement Measures for Model Predictions

Table 8 presents the Cohen’s Kappa and Matthews correlation coefficient (MCC) values for all the model configurations. For SNOMED CT, Fusion/Reranker achieved strong agreement (Kappa = 0.7829, MCC = 0.7885), outperforming BioClinicalBERT alone (0.7423/0.7511) and dense-only retrieval-augmented generation (0.6871/0.6998). The BM25 sparse retrieval configuration exhibited the lowest agreement (0.5751/0.5910), highlighting the limitations of traditional retrieval methods.

For LOINC, both Fusion/Reranker and dense-only retrieval-augmented generation (0.9773/0.9777) achieved near-perfect agreement, while BioClinicalBERT demonstrated high agreement (0.9318/0.9338). The BM25 configuration remained strong (0.9093/0.9124) but was inferior to the dense approaches.

For ICD-11, Fusion/Reranker showed substantial agreement (0.8435/0.8457), surpassing BioClinicalBERT (0.7840/0.7878) and dense-only retrieval-augmented generation (0.7856/0.7891). The BM25 configuration demonstrated moderate improvement over BioClinicalBERT alone (0.8049/0.8078) but remained below Fusion/Reranker.

These results confirm the following: (1) BM25 consistently underperforms compared to dense retrieval; (2) dense retrieval excels for standardized codes (LOINC) but requires fusion for complex mappings (SNOMED CT); and (3) Fusion/Reranker provides optimal agreement across all the terminologies.

### 3.9. Statistical Comparison Between Model Architectures

Table 9 presents statistical comparisons between the models using McNemar’s test and permutation testing. For SNOMED-CT, Fusion/Reranker showed a non-significant improvement over BioClinicalBERT alone (McNemar *p* = 0.500, Perm *p* = 0.491), with an accuracy difference of 0.0378, but significantly outperformed BM25 sparse retrieval (McNemar *p* = 0.0005, Perm *p* = 0.0002), with an accuracy difference of 0.2075.

For LOINC, Fusion/Reranker exhibited non-significant improvements over both BioClinicalBERT alone (McNemar *p* = 0.625) and the BM25 configuration (McNemar *p* = 0.250). For ICD-11, Fusion/Reranker demonstrated non-significant improvements over BioClinicalBERT alone (McNemar *p* = 0.375) and BM25 (McNemar *p* = 0.500). Further data and statistics are reported in Table 9.

All the McNemar *p*-values comparing BioClinicalBERT to Fusion/Reranker exceeded 0.375, indicating insufficient evidence for statistical superiority. This could be due also to the relatively small sample size (n = 53). However, the BM25 vs. Fusion/Reranker comparison for SNOMED-CT showed clear statistical significance (*p* < 0.001), demonstrating the superiority of dense embeddings over sparse retrieval for complex medical code mapping.

## 4. Discussion

This study developed and validated a hybrid pipeline integrating BioBERT for named entity recognition with BioClinicalBERT and retrieval-augmented generation to standardize anatomopathological reports across multiple ontologies. The system achieved excellent F1-scores for entity extraction and demonstrated substantial to near-perfect agreement for terminology mapping across SNOMED CT, LOINC, and ICD-11. These results confirm the technical feasibility of automated clinical entity extraction and standardization. However, statistical testing revealed that the augmentation benefits did not achieve significance at conventional thresholds given the current sample size.

### 4.1. BioBERT Performance for Named Entity Recognition

Our BioBERT model achieved a mean F1-score of 0.963 across the sample type (F1 = 0.97), test performed (F1 = 0.98), and finding (F1 = 0.93) entities, substantially exceeding the baseline performance thresholds. These results surpass the performance reported by Nath et al., who achieved F1-scores between 0.808 and 0.894 using BiLSTM-CRF architectures on i2b2 clinical datasets [39]. The substantial performance advantage demonstrates that transformer-based self-attention mechanisms capture long-range contextual dependencies more effectively than sequential recurrent architectures. Similarly, our results exceed the BioBERT performance reported by Lee et al., who achieved a maximum F1 of 0.898 on biomedical entity recognition benchmarks [16]. The higher performance in our study likely reflects task-specific fine-tuning on anatomopathological domain vocabulary, where consistent terminology usage and standardized report structures facilitate entity boundary detection.

Our precision of 0.969 and recall of 0.958 exceed the ranges of 0.84 to 0.87 reported for the SciBERT and BlueBERT variants [40], indicating that BioBERT pre-training on PubMed abstracts provides particularly effective initialization for medical entity extraction tasks. The test performed entity achieved the highest F1-score of 0.98, suggesting that standardized histological technique nomenclature exhibits less lexical variability than finding entities. The extremely high t-statistics for the precision, with *p* < 0.001, confirms the statistical significance, though the magnitude reflects the small sample size (n = 3), amplifying the test statistics. This suggests that procedural terminology benefits from greater standardization across pathology practice than diagnostic descriptions, which exhibit greater contextual variation and require deeper semantic understanding for accurate extraction.

### 4.2. Error Patterns and Entity Complexity

Error analysis revealed distinct patterns across the entity types, with the test performed exhibiting the highest relative error rate of 6.9% despite achieving the highest F1-score. This apparent contradiction reflects the large absolute token count for the test performed entities, where 16 misclassifications yield moderate error rates while maintaining high overall accuracy. The variety of expressions, abbreviations, and contextual descriptions for histological procedures creates disambiguation challenges that occasionally confuse the classification system. Common error sources included abbreviated technique names (e.g., “H&E” versus “hematoxylin and eosin”), procedural variations (e.g., “immunohistochemistry” versus “immunostaining”), and compound procedures described as single entities. In contrast, entity identification demonstrated the lowest relative error of 1% with only two misclassifications, indicating that diagnostic terminology follows more consistent patterns within anatomopathological reports. Sample type entities showed intermediate error characteristics, balancing the standardization of anatomical terminology with variations in specimen preparation.

The confusion matrix analysis revealed that most errors involved adjacent entity boundaries rather than complete entity category confusion, suggesting that the model effectively learns semantic distinctions but occasionally struggles with precise boundary identification in complex multi-word expressions. For example, phrases like “lymph node biopsy specimen” occasionally resulted in boundary errors between the sample type and the test performed entities. These error patterns align with observations from clinical named entity recognition studies showing that procedural and diagnostic concepts exhibit different linguistic regularities [39,41]. The bootstrap CIs confirm that these performance patterns represent stable model characteristics rather than artifacts of a particular test set composition.

### 4.3. Dataset Characteristics and Generalizability Considerations

While our focused corpus of 560 reports enabled high within-domain performance, several factors warrant consideration regarding generalizability. First, the dataset derives from a single institution, potentially with homogeneous reporting practices, terminology preferences, and pathologist writing styles. Multi-institutional studies have demonstrated that named entity recognition performance can decrease by 5–15% when models are applied to external datasets without retraining [42]. Our French-language corpus may limit the direct applicability to other languages, though recent work on multilingual biomedical models suggests that cross-lingual transfer learning can partially mitigate this limitation [43]. Second, anatomopathological reports exhibit a more standardized structure compared to other clinical note types, such as emergency department records or physician progress notes, potentially making our task somewhat easier than general clinical named entity recognition [44]. The finding entity demonstrated lower recall than the other entity types, likely reflecting the greater semantic diversity in diagnostic descriptions than in standardized specimen types and procedural nomenclature. This performance pattern aligns with findings from clinical entity recognition studies, which show that diagnostic concepts exhibit more variable expression than laboratory tests or anatomical structures [41]. Third, our corpus emphasizes general anatomic pathology cases, potentially under-representing rare diagnoses, complex syndromic presentations, or specialized pathology domains such as neuropathology or forensic pathology. External validation across multi-institutional datasets with diverse pathology subspecialties would provide a more robust assessment of generalizability. The narrow CIs for the precision suggest that the positive predictive value remains stable across bootstrap samples, while the wider recall intervals indicate greater sensitivity to the corpus composition.

### 4.4. Multi-Ontology Code Mapping Performance

Our Fusion/Reranker architecture achieved a macro-F1 of 0.7201 for the ICD-11 mapping, substantially exceeding the previously reported performance. Bhutto et al. achieved a macro-F1 of only 0.655 using deep recurrent convolutional neural networks with scaled attention on MIMIC-III ICD-10 coding tasks [45]. Chen et al. reported a macro-F1 around 0.701 using BioBERT and XLNet embeddings with rule-based approaches for ICD-10-CM coding [46]. Our superior performance, despite ICD-11’s larger label space (approximately 55,000 codes versus 14,000 in ICD-10-CM) and finer granularity, indicates that the combination of supervised classification with dense retrieval provides a more robust approach to handling rare codes and unseen concept variations than classification-only approaches. The retrieval component acts as a semantic buffer, allowing the system to map entities to terminology concepts even when training examples are sparse or absent. This addresses the fundamental challenge of medical code prediction: extreme class imbalance, where a small number of codes account for the majority of cases. In contrast, thousands of rare codes appear infrequently [47]. LOINC mapping achieved an exceptionally high macro-F1 of 0.9294 with a Cohen’s Kappa of 0.9773, indicating near-perfect agreement. This strong performance likely reflects the more constrained vocabulary space of laboratory test nomenclature compared to the broader concept coverage of SNOMED CT and ICD-11 [23]. Laboratory tests follow standardized naming conventions with limited synonyms, facilitating accurate code assignment through both classification and retrieval mechanisms. Additionally, our corpus likely contained fewer distinct LOINC codes than SNOMED CT or ICD-11, thereby reducing the complexity of the classification task. SNOMED CT mapping achieved a moderate macro-F1 of 0.6159 with a Cohen’s Kappa of 0.7829, suggesting challenges in handling the extensive concept hierarchy and semantic relationships within this comprehensive terminology [22]. The discrepancy between Cohen’s Kappa and macro-F1 likely reflects class imbalance effects, where frequent concepts achieve high accuracy while rare concepts contribute disproportionately to macro-F1 calculations. The MCC of 0.7885 for SNOMED CT confirms the substantial predictive quality when accounting for all the confusion matrix elements, including true negatives, which are typically abundant in multi-class problems with many infrequent classes.

### 4.5. Statistical Significance and Sample Size Considerations

The McNemar’s and permutation tests indicated that the observed performance improvements for Fusion/Reranker did not achieve statistical significance at the conventional α = 0.05 threshold. The most probable explanation for this is the limited sample size of 53 test instances, which provides insufficient statistical power to detect moderate effect sizes. A post hoc power analysis suggests that detecting a 5% accuracy difference with 80% power at α = 0.05 would require approximately 200–250 test samples, given the observed variance [48]. The higher descriptive performance of Fusion/Reranker across all the metrics suggests potential clinical utility, despite the lack of statistical significance. For SNOMED CT, the accuracy improvement represents meaningful gains when extrapolated to large-scale coding operations processing thousands of reports monthly. A hospital processing 500 pathology reports daily would see approximately 9–10 additional correctly coded reports per day with the Fusion/Reranker architecture. The Cohen’s Kappa values demonstrated substantial to near-perfect agreement for all the models, with Fusion/Reranker consistently achieving higher agreement levels. The practical implications of these findings suggest that retrieval-augmented generation provides measurable performance gains that may translate to clinical value. Consequently, while the current results demonstrate a consistent and promising trend, they underscore the critical need for validation on larger-scale, multi-institutional datasets to conclusively establish the statistical superiority and generalizability of the Fusion/Reranker approach.

### 4.6. Retrieval-Augmented Generation Integration

The integration of dense retrieval with supervised classification represents a hybrid approach that combines the complementary strengths of both paradigms. Supervised classification effectively learns discriminative patterns from labeled training data, achieving high accuracy for frequent entity–code pairs with abundant training examples [49]. The classifier learns to recognize linguistic patterns associated with specific codes, such as associating “adenocarcinoma” mentions with particular ICD-11 oncology codes. However, the classification performance degrades for rare codes with few training instances (e.g., those that appear only once or twice) and fails for unseen concepts absent from the training data. This limitation is particularly problematic in medical coding, where the Zipfian distributions mean that many codes are extremely rare [47]. Retrieval-augmented generation addresses these limitations by grounding the predictions in external knowledge bases that provide comprehensive coverage of terminology [29,30]. The semantic similarity search enables mapping to appropriate codes even when exact training examples are unavailable, thereby improving the robustness to the long-tail distributions characteristic of medical terminologies. For instance, if the training set lacked examples of a specific rare tumor subtype, the retrieval component could still identify the correct code by matching the entity description to similar concepts in the terminology database. Recent studies involving biomedical question answering and clinical reasoning tasks have demonstrated that retrieval-augmented approaches improve factual accuracy and reduce hallucinations compared to generation-only systems [50]. Our fusion mechanism learned to balance classification confidence with retrieval similarity through cross-validation, optimizing the trade-off between discriminative accuracy and semantic robustness. The learned weights effectively route decisions to the more reliable component depending on the input characteristics, entity types, and terminology structures. For LOINC, the classification component demonstrated sufficient accuracy to indicate that retrieval provided minimal additional benefit, as reflected in the small performance gap between BioClinicalBERT and Fusion/Reranker. In contrast, for ICD-11, with its larger label space and greater semantic complexity, retrieval augmentation provided more substantial improvements by retrieving appropriate codes for rare diagnostic concepts where training examples were sparse.

### 4.7. Agreement Measures Interpretation

Cohen’s Kappa and MCC provide complementary perspectives on model–annotation agreement by accounting for chance concordance and imbalanced class distributions [37]. For LOINC, the near-perfect Kappa of 0.9773 and MCC of 0.9777 indicate that the model achieves agreement levels approaching human-level performance on this structured laboratory test nomenclature. Inter-annotator agreement studies for LOINC coding typically report Kappa values between 0.85 and 0.98 [51], suggesting that our automated system performs comparably to human coders. The slight discrepancy between Kappa and MCC reflects the different weighting schemes for the confusion matrix elements, with MCC providing a balanced measure across all the prediction categories, including true negatives. For SNOMED CT, the substantial Kappa of 0.7829 indicates strong agreement despite a moderate macro-F1 of 0.6159, reflecting the impact of frequent concept classes achieving high accuracy. In contrast, rare concepts contribute disproportionately to macro-F1 calculations. This pattern is common in imbalanced classification tasks where the accuracy can be high while the macro-averaged metrics remain moderate due to poor performance on infrequent classes. The ICD-11 Kappa of 0.8435 represents moderate to substantial agreement according to standard interpretation guidelines [37], indicating clinically useful performance that nevertheless requires human verification for quality assurance. The inter-annotator agreement for ICD-11 coding typically ranges from 0.60 to 0.85, depending on the code granularity and specialty domain [52], suggesting our automated system performs within the range of human coder variability. The consistency of agreement patterns across BioClinicalBERT, retrieval-augmented generation, and Fusion/Reranker confirms that different architectural approaches achieve similar levels of concordance, with hybrid fusion providing incremental improvements. These agreement measures provide more clinically interpretable performance assessments than raw accuracy, particularly for imbalanced medical coding tasks, where the majority-class prediction can achieve superficially high accuracy while failing to capture rare diagnostic concepts.

### 4.8. Clinical Implications and Deployment Considerations

Automated extraction and standardization of anatomopathological entities provide multiple clinical benefits, including improved data quality, enhanced interoperability, accelerated research capabilities, and reduced manual coding burden [53]. Standardized terminologies enable semantic queries across institutional databases, supporting comparative effectiveness research, epidemiological surveillance, and clinical decision support systems [54]. The high LOINC mapping accuracy facilitates the integration of laboratory tests with electronic health records, enabling automated result retrieval and longitudinal tracking of specific biomarkers or tumor markers across patient encounters. ICD-11 standardization supports epidemiological reporting to public health agencies and allows accurate quantification of the disease burden for healthcare resource allocation decisions [24]. SNOMED CT mapping provides a granular representation of clinical concepts, supporting interoperability between heterogeneous healthcare information systems [22]. However, several considerations must be addressed before clinical deployment. First, the model outputs require validation by qualified pathologists before incorporation into official medical records, as coding errors could impact clinical decisions, billing accuracy, reimbursement, and quality metrics used for hospital performance evaluation. A graduated deployment approach could begin with high-confidence predictions (e.g., model confidence > 0.95) receiving automatic approval, while uncertain cases are routed to manual review. Second, ongoing model monitoring is essential to detect performance degradation when encountering evolving terminology, novel pathology entities, or shifting documentation practices. Concept drift in medical language occurs as new diagnostic techniques emerge and terminology evolves [55]. Third, transparency mechanisms such as confidence scores, retrieval evidence, and explanation interfaces should accompany predictions to support human verification workflows and build trust among pathologist users. Fourth, the system must integrate seamlessly with existing pathology information systems, requiring attention to standards, including Health Level Seven International (HL7) messaging standards, LOINC interoperability specifications, and integration points with laboratory workflows.

### 4.9. Computational Efficiency and Scalability

The transformer-based architecture requires substantial computational resources during both the training and inference phases, with the BioBERT and BioClinicalBERT models containing approximately 110 million parameters each. GPU acceleration proved essential for achieving acceptable training times, with our three-epoch named entity recognition fine-tuning requiring approximately 6–8 h on NVIDIA Tesla V100 GPUs. Training the standardization model (10 epochs) required approximately 4–5 h on similar hardware. The inference latency for processing individual reports averaged 2–3 s per document, including entity extraction and multi-ontology standardization, representing acceptable performance for batch processing applications but potentially limiting real-time interactive use cases. For comparison, manual coding of pathology reports by humans typically takes 3–5 min per report [54], suggesting that, even with the computational overhead, automated approaches offer substantial time savings. The retrieval-augmented generation component adds to the computational overhead through the dense vector similarity search across terminology databases containing hundreds of thousands of concepts. We employed the approximate nearest neighbor search using FAISS indexing (version 1.8.0 (Meta Platforms Inc., Menlo Park, CA, USA)) to maintain sub-second retrieval latency [56]. FAISS enables efficient similarity searching through product quantization and inverted-file indexing, reducing the search time from linear O(n) to sublinear. For large-scale deployment processing thousands of daily reports, a distributed computing infrastructure would enable parallel processing across multiple GPU nodes. Model quantization techniques (e.g., INT8 quantization) and knowledge distillation could reduce the computational requirements by 2–4× while maintaining acceptable accuracy decreases of typically 1–3% [57]. These optimization techniques would enable deployment on more modest hardware configurations, potentially allowing inference on CPU-only systems for smaller institutions. The batch size of eight during training represents a balance between GPU memory constraints (16 GB for V100) and gradient estimation quality, with larger batches potentially improving the convergence stability at the cost of increased memory consumption.

### 4.10. Limitations

Several methodological limitations warrant acknowledgment. First, while our pipeline currently treats entity recognition (via BioBERT) and terminology normalization (via retrieval-augmented generation) as separate stages, recent studies suggest that integrating terminology-aware supervision can enhance the alignment of representation spaces. Jin et al. [58] demonstrated that high-confidence pseudo-labels improve feature discrimination in foundation models, a principle that can be applied to text based entity linking. Similarly, Lin et al. [59] utilized restrictive and contrastive decoding to guide large language models toward valid biomedical entities without the need for complete retraining. These studies highlight complementary strategies for improving semantic alignment and motivate future efforts to jointly optimize entity representations and terminology mapping. Second, the integration of multiple clinical terminologies (SNOMED CT, LOINC, ICD-11) presents a fundamental semantic challenge. These systems are modeled along different primary axes, LOINC for laboratory observations, SNOMED CT for comprehensive clinical concepts, and ICD-11 for disease classification. In our pathology-specific implementation, we map these broader axes to our entity types: SNOMED CT for sample type (specimens), LOINC for test performed (methods), and a combination of SNOMED CT and ICD-11 for finding (diagnoses). This task-specific routing mitigates direct conflicts from a naive merge but does not fully resolve the underlying discrepancies in granularity. For instance, mapping a precise histopathological finding to a broader ICD-11 category may incur a loss of specificity. Future work could leverage unified biomedical ontologies, such as the UMLS Metathesaurus, to enable more coherent cross-terminology alignment. Third, the dataset derives from a single institution in Tunisia, potentially limiting the generalizability to other geographic regions, healthcare systems, or pathology subspecialties with different reporting conventions. French-language medical terminology exhibits regional variations between France, Belgium, Switzerland, and North African countries, which could affect the model performance on reports from other French-speaking regions [60]. Multi-institutional validation across diverse clinical settings is necessary to assess external validity. Fourth, the test set size of 53 samples provided insufficient statistical power to detect moderate effect sizes between model architectures, as evidenced by the non-significant McNemar and permutation test results despite the descriptive performance differences. Sample size calculations suggest that 200–250 test samples would be required for adequately powered comparative evaluations [48]. Fifth, manual annotation by pathologists, while establishing gold-standard labels, may introduce inter-rater variability that affects ceiling performance estimates. We did not conduct a formal inter-annotator agreement assessment with multiple annotators, which would quantify the annotation consistency and provide more robust ground truth labels. Sixth, the corpus emphasizes general anatomic pathology cases, potentially under-representing rare diagnoses, complex syndromic presentations, or specialized pathology domains such as neuropathology, forensic pathology, or molecular pathology. Seventh, the computational resource requirements for transformer-based architectures may limit the deployment feasibility in resource-constrained healthcare settings without adequate GPU infrastructure, though the aforementioned optimization techniques could mitigate this limitation. Eighth, this study evaluated the performance on retrospective data without prospective validation in real-world clinical workflows, where integration challenges, user acceptance factors, and workflow disruption may impact the practical utility. Finally, we did not evaluate the cost-effectiveness or time savings compared to manual coding, which would be essential for healthcare administrators considering implementation.

## 5. Conclusions

This study demonstrates that BioBERT-based named entity recognition, combined with BioClinicalBERT and retrieval-augmented generation, achieves strong performance in automated extraction and multi-ontology standardization of anatomopathological report entities. The hybrid architecture achieved excellent F1-scores as well as mean precision and recall, as confirmed by the bootstrap CIs and statistical significance testing, demonstrating performance substantially exceeding the baseline thresholds. Multi-ontology standardization demonstrated substantial to near-perfect agreement across the SNOMED CT, LOINC, and ICD-11 terminologies, with particularly high performance for the LOINC laboratory test mapping. While retrieval-augmented generation provided descriptive performance improvements across all the tasks, with consistent gains in the accuracy, precision, recall, and F1-scores, statistical testing revealed non-significant differences given the current sample size of 53 test instances, indicating that larger validation studies are necessary to establish augmentation benefits with adequate statistical power definitively. The system demonstrates preliminary practical feasibility for reducing the manual coding burden, improving the data quality, and enhancing the semantic interoperability across healthcare institutions. However, several critical steps remain before clinical deployment can be recommended. Multi-institutional validation studies across diverse geographic regions, languages, and pathology subspecialties are essential to assess the generalizability and identify potential performance degradation scenarios when encountering different documentation styles, terminology preferences, and case mix patterns. A prospective evaluation comparing automated coding with expert pathologist annotations in real-world workflow conditions would quantify the accuracy, identify failure modes, and establish quality assurance protocols for clinical integration. Integration with existing laboratory information systems and electronic health record platforms requires attention to computational efficiency, latency constraints, inference scalability, and failure mode handling to ensure reliable operation in production environments without disrupting clinical workflows. Human-in-the-loop workflows with confidence-based routing to manual review can maintain quality standards while maximizing automation benefits, optimizing the balance between efficiency gains and clinical safety through adaptive thresholding based on validation studies.

Future research directions include expansion to multilingual pathology corpora enabling cross-linguistic model transfer through multilingual BERT variants, incorporation of multimodal data such as structured report sections and histopathological images for comprehensive diagnostic coding, hierarchical code prediction exploiting the terminology structure and semantic relationships to improve rare code detection, active learning approaches to continuously improve performance with minimal annotation burden through intelligent sample selection, and federated learning frameworks enabling collaborative model development across institutions while preserving data privacy and regulatory compliance. The demonstrated technical feasibility, combined with the straightforward clinical utility for improved data standardization, enhanced research capabilities, reduced coding costs, and better interoperability, motivates continued development toward robust, validated systems supporting pathology informatics infrastructure in modern healthcare systems, ultimately advancing the transformation of unstructured clinical narratives into actionable structured knowledge, supporting evidence-based medicine, precision health initiatives, and data-driven clinical decision support.

## Figures and Tables

**Table 1:** Annotation guidelines and examples for entity types.

Entity Type	Positive Examples	Negative Examples
Sample Type	Biopsy of bone, Biopsy of thyroid, Bronchoalveolar lavage fluid specimen, Biopsy of prostate	Osteosarcoma (Finding), Immunohistochemistry (Test Performed)
Test Performed	Immunohistochemistry, Biochemistry, Dermatopathology	Hashimoto’s thyroiditis (Finding), Biopsy of thyroid (Sample Type)
Finding	Osteosarcoma, Mesothelioma, COVID-19, Melanoma in situ	Histopathology (Test Performed), Bone marrow sample (Sample Type)

**Table 2:** Performance metrics for the BioBERT model on the test set, organized by entity.

Entity	Precision	Recall	F1-Score	Support
Sample Type	0.97	0.98	0.97	192
Test Performed	0.97	0.99	0.98	554
Finding	0.97	0.90	0.93	231

**Table 3:** Absolute and relative errors per entity for the BioBERT model.

Entity	Absolute Error	Relative Error
Sample Type	14	0.025
Test Performed	16	0.069
Finding	2	0.010

**Table 4:** The 95% confidence intervals (CIs) for the precision, recall, and F1-score estimated via bootstrapping.

Metric	Precision	Recall	F1-Score
95% CI	0.967–0.970	0.900–0.995	0.933–0.982

**Table 5:** One-sample *t*-test results comparing the model performance against a baseline of 0.5.

Metric	T (Approx.)	*p*-Value
Precision	612.029	<0.0001
Recall	15.71	0.004
F1-Score	30.24	0.0011

**Table 6:** Metric statistics by label.

Metric	Mean	Standard Deviation (Std)
Precision	0.969	0.001
Recall	0.958	0.050
F1-Score	0.963	0.025

**Table 7:** Descriptive performance metrics for all the models and tasks (N = 53).

Label	Model	Accuracy	Precision Macro	Recall Macro	F1-Score Macro
SNOMED CT Code	BioClinicalBERT only	0.7547	0.5881	**0.6667**	0.6124
	RAG dense only	0.6981	0.5833	0.5960	0.5856
	BioClinicalBERT + BM25 (sparse RAG)	0.5849	0.5000	0.4907	0.4944
	Fusion/Reranker (BioClinicalBERT + RAG dense)	**0.7925**	**0.6136**	0.6263	**0.6159**
LOINC Code	BioClinicalBERT only	0.9434	0.8284	0.8824	0.8445
	RAG dense only	0.9811	0.9216	0.9412	0.9294
	BioClinicalBERT + BM25 (sparse RAG)	0.9245	0.7963	0.8333	0.8056
	Fusion/Reranker (BioClinicalBERT + RAG dense)	**0.9811**	**0.9216**	**0.9412**	**0.9294**
ICD-11 code	BioClinicalBERT only	0.7925	0.6623	0.7171	0.6772
	RAG dense only	0.7925	0.6500	0.6875	0.6583
	BioClinicalBERT + BM25 (sparse RAG)	0.8113	0.6813	0.7125	0.6880
	Fusion/Reranker (BioClinicalBERT + RAG dense)	**0.8491**	**0.7115**	**0.7500**	**0.7201**

**Table 8:** Cohen’s Kappa and Matthews correlation coefficient (MCC) for all the models and tasks (N = 53).

Label	Model	Cohen’s Kappa	MCC
SNOMED CT code	BioClinicalBERT only	0.7423	0.7511
	RAG dense only	0.6871	0.6998
	BioClinicalBERT + BM25 (sparse RAG)	0.5751	0.5910
	Fusion/Reranker	**0.7829**	**0.7885**
LOINC code	BioClinicalBERT only	0.9318	0.9338
	RAG dense only	0.9773	0.9777
	BioClinicalBERT + BM25 (sparse RAG)	0.9093	0.9124
	Fusion/Reranker	**0.9773**	**0.9777**
ICD-11 code	BioClinicalBERT only	0.7840	0.7878
	RAG dense only	0.7856	0.7891
	BioClinicalBERT + BM25 (sparse RAG)	0.8049	0.8078
	Fusion/Reranker	**0.8435**	**0.8457**

**Table 9:** Statistical comparison between models (McNemar, accuracy difference, and permutation tests, N = 53).

Label	Comparison	McNemar *p*	Accuracy Difference	Perm *p*
SNOMED CT code	BioClinicalBERT only vs. Fusion/Reranker	0.500	0.0378	0.4911
	BioClinicalBERT + BM25 (sparse RAG) vs. Fusion/Reranker	0.0005	0.2075	0.0002
LOINC code	BioClinicalBERT only vs. Fusion/Reranker	0.625	0.0377	0.6171
	BioClinicalBERT + BM25 (sparse RAG) vs. Fusion/Reranker	0.250	0.0566	0.243
ICD-11 code	BioClinicalBERT only vs. Fusion/Reranker	0.375	0.0566	0.3721
	BioClinicalBERT + BM25 (sparse RAG) vs. Fusion/Reranker	0.500	0.0377	0.487

## Data Availability

The data that support the findings of this study are openly available upon request from the corresponding author.
